# Supplementary material for: Butyrylcarnitine Elevation in Newborn Screening: Reducing False Positives and Distinguishing between Two Rare Diseases through the Evaluation of New Ratios
Source: Biomedicines. 2023 Dec 7;11(12):3247. doi: 10.3390/biomedicines11123247 (PMC10741594; doi:10.3390/biomedicines11123247)
Supplement: Supplementary file 1 [file biomedicines-11-03247-s001.zip › biomedicines-2679277-supplementary.pdf]

Table S1. Panel of the disorders screened in the Italian mandatory screening program.

**Disorder**	**Abbreviation**	**Marker**
Phenylketonuria	PKU	Phe
Benign hyperphenylalaninemia	H-PHE	Phe
Biopterin defect in cofactor biosynthesis	BIOPT (BS)	Phe
Biopterin defect in cofactor regeneration	BIOPT (REG)	Phe
Tyrosinemia type I	TYR I	Tyr
Tyrosinemia type II	TYR II	Tyr
Maple syrup urine disease (MSUD)	MSUD	Val; Ile + Leu
Homocystinuria (CBS deficiency)	HCY	Met
Homocystinuria (severe MTHFR deficiency)	MTHFR	Met
Glutaric acidemia type I	GA I	C5DC
Isovaleric acidemia	IVA	C5
Beta-ketothiolase deficiency	BKT	C5:1; C5OH
3-hydroxy-3-methylglutaric acidemia	HMG	C5OH; C6DC
Propionic acidemia	PA	C3
Methylmalonic acidemia (Mut)	MUT	C3
Methylmalonic acidemia (A)	CbI A	C3
Methylmalonic acidemia (B)	CbI B	C3
Methylmalonic acidemia with homocystinuria (CbI C)	CbI C	C3
Methylmalonic acidemia with homocystinuria (CbI D)	CbI D	C3
2 methylbutyryl CoA dehydrogenase deficiency	2MBG	C5
Malonic acidemia	MAL	C3DC
Multiple carboxylase deficiency	MCD	C5OH
Citrullinemia type I	CIT	Cit
Citrullinemia type II	CIT II	Cit
Argininosuccinic acidemia	ASA	Cit
Carnitine transporter defect	CUD	C0
Carnitine palmitoyl transferase 1A deficiency	CPT I	C0
Carnitine-acylcarnitine translocase deficiency	CACT	C16; C18:2; C18:1; C18
Carnitine palmitoyltransferase II deficiency	CPT II	C16; C18:2; C18:1; C18
Long chain 3-hydroxyacyl-CoA dehydrogenase deficiency	VLCAD	C14:1; C14:2; C14
Trifunctional protein deficiency	TFP	C16:1OH C16OH; C18:1OH; C18OH
Long chain 3-hydroxyacyl-CoA dehydrogenase deficiency	LCHAD	C16:1OH; C16OH; C18:1OH; C18OH
Medium chain acyl-CoA dehydrogenase deficiency	MCAD	C6; C8; C10:1; C10
Medium- and short-chain-3-hydroxyacyl-CoA dehydrogenase deficiency	M/SCHAD	C4-OH
Glutaric acidemia type II	GA2/MADD	C4-C18 saturated, unsaturated
Tyrosinemia type III	TYR III	Tyr
Glycine N-methyltransferase deficiency	GNMT	Met
Methionine adenosyltransferase deficiency	MAT	Met
S-adenosylhomocysteine hydrolase deficiency	SAHH	Met
3-methylglutaconic acidemia	3MGCA	C5-OH
3-methylcrotonyl-CoA carboxylase deficiency	3MCC	C5-OH
2-methyl-3-hydroxybutyryl-CoA dehydrogenase deficiency	2M3HBA	C5:1; C5-OH
Isobutyryl-CoA dehydrogenase deficiency	IBG	C4
Short-chain acyl-CoA dehydrogenase deficiency	SCAD	C4

Table S2. List of ratios investigated.

Number	Ratio	Name of analyte in denominator
1	C4/C0	Free carnitine
2	C4/C2	Acetylcarnitine
3	C4/C3	Propionylcarnitine
4	C4/C3DC\C4OH	Malonyl\3-hydroxybutyrylcarnitine
5	C4/C4DC\C5OH	Methylmalonyl\3-hydroxyisovalerylcarnitine
6	C4/C5	Isovalerylcarnitine
7	C4/C5:1	Tiglylcarnitine
8	C4/C5DC\C6OH	Glutaryl\3-hydroxyhexanoylcarnitine
9	C4/C6	Hexanoylcarnitine
10	C4/C6DC	Adipylcarnitine
11	C4/C8	Octanoylcarnitine
12	C4/C8:1	Octenoylcarnitine
13	C4/C10	Decanoylcarnitine
14	C4/C10:1	Decenoylcarnitine
15	C4/C10:2	Decadienoylcarnitine
16	C4/C12	Dodecanoylcarnitine
17	C4/C12:1	Dodecenoylcarnitine
18	C4/C14	Myristoylcarnitine
19	C4/C14:1	Tetradecenoylcarnitine
20	C4/C14:2	Tetradecadienoylcarnitine
21	C4/C14OH	3-hydroxytetradecanoylcarnitine
22	C4/C16	Palmitoylcarnitine
23	C4/C16:1	Hexadecenoylcarnitine
24	C4/C16OH	3-hydroxyhexadecanoylcarnitine
25	C4/C16:1OH\C17	3-hydroxyhexadecadecenoyl\heptadecanoylcarnitine
26	C4/C18	Stearoylcarnitine
27	C4/C18:1	Oleylcarnitine
28	C4/C18:2	Octadecadienoylcarnitine
29	C4/C18OH	3-hydroxyoctadecanoylcarnitine
30	C4/C18:1OH	3-hydroxyoctadecenoylcarnitine
31	C4/C18:2OH	3-hydroxyoctadecadienoylcarnitine
32	C4/C20	Eicosanoylcarnitine
33	C4/C22	Docosanoylcarnitine
34	C4/C24	Tetracosanoylcarnitine
35	C4/C26	Hexacosanoylcarnitine

Table S3. Diagnosis performed from December 2017 to December 2022.

Disease	Diagnosis
Propionic Acidemia	1
Glutaric Acidemia Type I	1
Citrullinemia Type I	3
Phenylketonuria	9
Hyperphenylalaninemia	25
Isovaleric Acidemia	2
2-MetyilbutyrylCoA Dehydrogenase deficiency	1
Methylmalonic Acidemia with Homocystinuria	1
Short Chain AcylCoA Dehydrogenase deficiency	26
IsobutyrylCoA Dehydrogenase deficiency	4
3-methylcrotonylcarboxylase Deficiency	5
Glutaric Acidemia Type II	1
Long Chain 3-OH-AcilCoA Dehydrogenase Deficinecy	1
S-adenosylhomocysteine hydrolase	1
X-ALD*	1
Hyperprolinemia*	2
Sibling Short Chain AcylCoA Dehydrogenase deficiency	1
Paternal Short Chain AcylCoA Dehydrogenase deficiency	2
Sibling Citrullinemia Type I	1
Maternal B12 deficiency	9
Maternal 3-methylcrotonylcarboxylase Deficiency	5

* diagnosis non included in the mandatory screening panel, performed after a signed parental consent.
